# Climate change adaptation networks for small and medium-sized cities

**DOI:** 10.1007/s43545-021-00267-7

**Published:** 2021-10-22

**Authors:** Simone Häußler, Wolfgang Haupt

**Affiliations:** 1 Aalen University, Electrical Engineering - Renewable Energies, Anton-Huber-Straße 25, 73430 Aalen, Germany; 2 Leibniz-Institute for Research on Society and Space, Flakenstraße 29-31, 15537 Erkner, Germany

**Keywords:** City networks, Climate change adaptation, Key actors, Knowledge transfer, Regional network framework, Participatory action research

## Abstract

Cities are particularly vulnerable to the impacts of climate change. Many larger cities have identified the potential impacts of different climate change adaptation scenarios. However, their smaller and medium-sized counterparts are often not able to address climate risks effectively due to a lack of necessary resources. Since a large number of cities worldwide are indeed small and medium-sized, this lack of preparedness represents a crucial weakness in global response systems. A promising approach to tackling this issue is to establish regional municipal networks. Yet, how might a regional network for small and medium-sized cities be systematically designed and further developed? Focussing on the German federal state of Baden-Wuerttemberg, we have explored this question by applying a participatory action research approach. As part of our research, we established a regional network framework for small and medium-sized cities. The framework supports small and medium-sized cities in identifying key regional actors, while taking local and regional contextual factors into account. Based on our findings, we suggest that other small and medium sized cities follow these steps: develop the knowledge base; build the network; and transfer and consolidate knowledge.

## Introduction

For many regions worldwide, climate change is leading to an increased intensity and frequency of extreme weather events (Dannenberg et al. 2019; Eckstein et al. 2021). The effects of climate change pose major challenges, especially to cities (Bulkeley 2010; Wamsler et al. 2013; Drolet and Sampson 2017; Palermo et al. 2020). Depending on their specific geographical location, cities are particularly vulnerable (Rosenzweig et al. 2010; Carter 2011) to severe heat waves, due to the urban heat island effect (Menberg et al. 2013; Jiricka-Pürrer et al. 2020), droughts (Kiem and Austin 2013; Guerreiro et al. 2018) and floods (Eckersley et al. 2018; Heidenreich et al. 2020; Dillenardt et al. 2021). Municipal climate adaptation therefore aims to provide cities with concepts and strategies to address and prepare for current and future climate risks, so that threats are avoided or reduced, as far as possible (Jolk 2015).

Particularly, small and medium-sized cities (hereinafter SMCs) with fewer than 100,000 inhabitants (BBSR 2020) often have very limited resources to identify local climate risks and build appropriate adaptation capacities (Otto et al. 2021). It is therefore highly important to examine the activities of such cities to identify the extent to which they will be able to address climate change (Giffinger et al. 2007; Bell and Jayne 2009).

Some SMCs involve themselves in municipal climate networks to support their adaptation efforts (Hauge et al. 2019; Papin 2019; Landauer et al. 2019; Goh 2020). Heikkinen et al. define municipal climate networks as *[…] organisations that aim to support cooperation between cities to improve their climate change mitigation and adaptation work* (2020). Municipal climate networks are formal, self-governed organisations and have formal structures, such as a head office and a staff of employees (Kern and Bulkeley 2009; Busch 2015; Haupt and Coppola 2019). The most visible networks consist of cities from various countries.

Busch (2015) and Haupt and Coppola (2019) refer to these internationally operating networks as transnational municipal (climate-) networks. Well-known examples are for instance *United Cities and Local Governments* (UCLG), *Local Governments for Sustainability* (ICLEI), *100 Resilient Cities* and *C40* (Labaeye and Sauer 2013; Haupt and Coppola 2019). Most of them, particularly the two latter ones, heavily focus on capital cities and large cities (Hunt and Watkiss 2011; Araos et al. 2016; Gordon 2016; Coppola et al. 2020; Haupt et al. 2020).

SMCs, on the other hand, are significantly less involved in international networks (Haupt and Coppola 2019; Kern 2019). They urgently need support as they often have very limited capacities for action or to pioneer and test their own place-based approaches. More precisely, SMCs need regionally and locally tailored solutions (Granberg et al. 2019). This could be facilitated through the development of regional climate adaptation networks (Stiller and Meijerink 2016; Schmid et al. 2016). A regional network of SMCs could help to form a community that requires each member to input fewer resources and benefit from greater synergies with other participating actors when seeking to address common problems.

The aim of our work is to develop a transferable framework for the initiation and establishment of regional networks for climate change adaptation in SMCs. In this respect, we first determine which key actors most effectively support adaptation measures in SMCs (hereinafter question 1). Next, we question how a regional network for SMCs can be systematically designed and established (hereinafter question 2). Our study is based on the hypothesis that regional networks for municipal climate adaptation can effectively promote climate change adaptation practices in SMCs.

Applying a participatory action research (PAR) approach, we established a regional network for municipal climate adaptation in the German state of Baden-Wuerttemberg in 2020. The network organised and hosted a region-wide workshop. We developed our framework based on manifold interactions with the municipal climate action officers that actively participated in this workshop.

### Case selection and case description

**Fig. 1:**
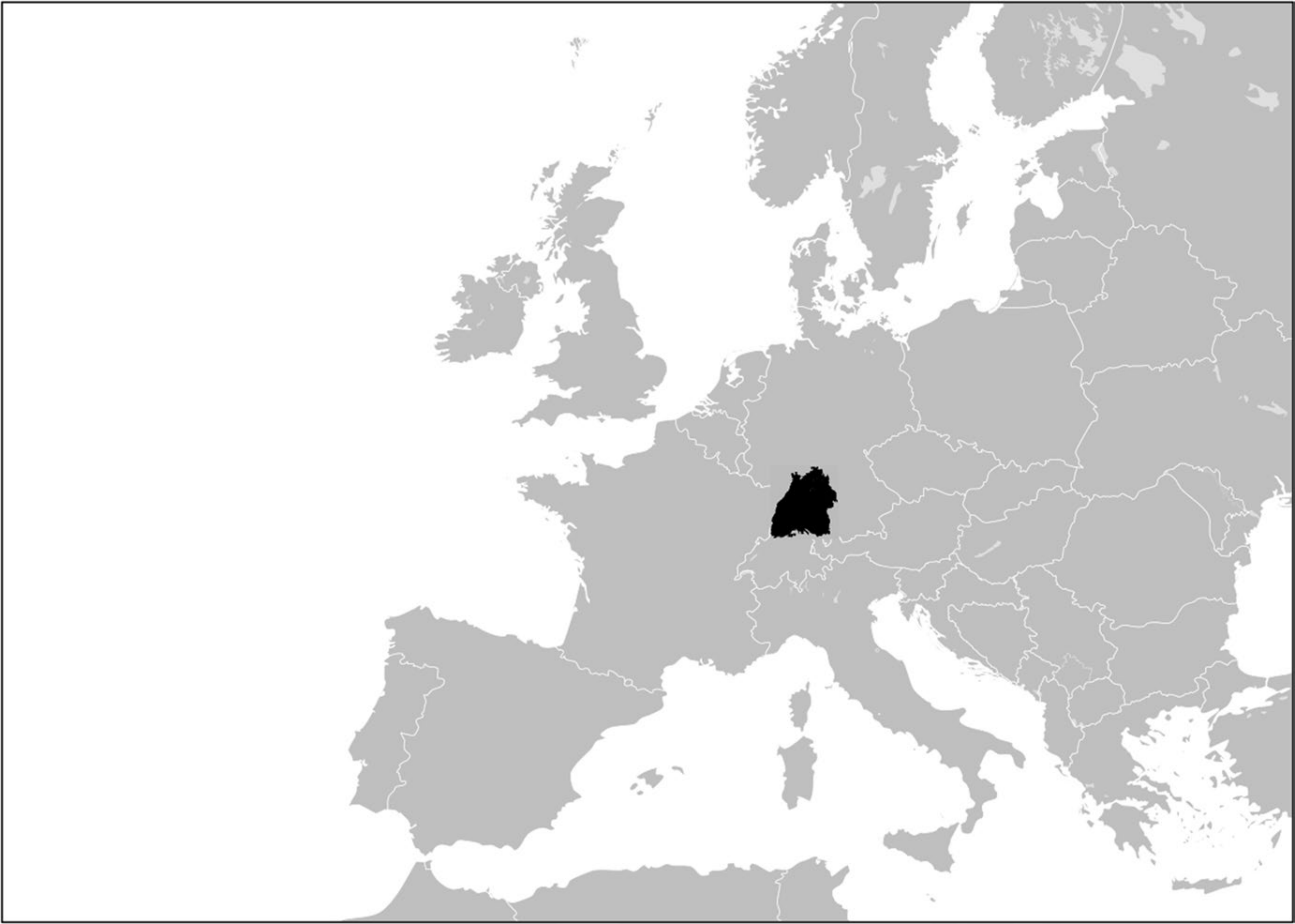
The location of the German state of Baden-Wuerttemberg on the map of Europe

We chose the south-west German federal state (*Bundesland*) of Baden-Wuerttemberg (Fig. 1) as our focus to demonstrate how municipal climate adaptation practices can be substantially supported by regional networks.

Due to its geographical location on the *Upper Rhine Graben*, Baden-Wuerttemberg is highly vulnerable to the effects of climate change, such as a steady increase in the number of very hot days and extreme drought (Scholze et al. 2020). The Rhine Graben stretches for a total of 300 km with a width of 30–40 km through Switzerland, France and Germany. It is known for extreme temperature developments along its edges (Harlé et al. 2019). Heat waves have occurred in Baden-Wuerttemberg in the summer months in recent years with high frequency and intensity (Rebetez et al. 2006; Herrmann and Sauerborn 2018). In the years 2003 and 2015, high temperatures and high humidity over long periods of time lead to extreme heat stress, which resulted in a significant increase of the heat-related mortality rate (Muthers et al. 2017).

With a population of approx. 11 million, Baden-Wuerttemberg is the third largest federal state of Germany. Within Europe, its population can be compared to midsize countries like Sweden or Austria. The population of Baden-Wuerttemberg is comparable to that of an American US-state such as Pennsylvania or Ohio. It extends over 35,751 km^2^ and is characterised by different geographical landscapes, such as the mountainous Black Forest (Feldberg summit 1493 m a.s.l.) and the Swabian Jura (around 500 m a.s.l.), with different climate risks (Hackenbruch et al. 2017).

In a comparison of Baden-Wuerttemberg with Germany, Sweden and Austria, it is noticeable that the percentage of inhabitants in large cities is remarkably low. Only one in five inhabitants live in one of the nine large cities of the federal state. Eight of these large cities remain significantly below the 500,000-population mark (Destatis 2020). Only the state capital Stuttgart exceeds this mark with approx. 609,000 inhabitants (ibid.). Stuttgart's particular climate vulnerability is caused by its sink-like basin location (Fallmann et al. 2014; Ketterer and Matzarakis 2014). To address this problem, Stuttgart has developed a climate adaptation strategy (Hebbert and Webb 2012). But above all, the state capital also has more resources and greater capacities for action than any of the other approx. 1100 municipalities in Baden-Wuerttemberg. Two of the other larger cities within the federal state, Freiburg and Heidelberg, have received numerous awards in recent years for their climate policies and sustainability activities. The city of Freiburg has developed and implemented professional green city branding strategies (Carter 2011; Mössner 2015). Moreover, Heidelberg is one of the few non-megacities to have been accepted into the global pioneer network *C40* (Lee and van de Meene 2012). Cities that are pioneering climate policies often have the ambition to attract followers and are referred to as climate policy leaders in academic literature (Liefferink and Wurzel 2018; Wurzel et al. 2019). Heidelberg and Freiburg are such climate policy leaders (Jänicke and Wurzel 2019; Otto et al. 2021). But they are unlikely to find many followers in Baden-Wuerttemberg as their approaches are not easily replicable for SMCs (Haupt 2020). In fact, most SMCs in Baden-Wuerttemberg (as well as in whole Germany) have not yet developed climate adaptation strategies or implemented substantial climate adaptation measures (Otto et al. 2021). This is concerning, because they are home to roughly 80% of Baden-Wuerttembergs population (Destatis 2020).

Nevertheless, these SMCs will also be increasingly affected by the impacts of climate change. Therefore, there is an urgent need for the development of regionally organised learning networks for SMCs. The scholarly debate (e.g. Matisoff and Edwards 2014; Fisher 2014; Stead and Pojani 2018; Haupt 2020) also suggests that cities with similar size and institutions can more easily learn from each other than cities that are drastically different from one another.

## Materials and methods

The social psychologist Kurt Lewin is often referred to as the *originator of action research* (Adelman 1993). Lewin first described his findings from field interviews with workers in his publication *Action research and minority problems* (1946). While there is no single definition of action research (AR henceforth), there are different approaches to describe this method. Hult and Lennung define it as a method that *simultaneously assists in practical problem-solving and expands scientific knowledge* (1980). According to Altrichter et al., AR is an *enquiry with people, rather than research on people* (2002). Van Buuren et al. add that AR *essentially is a matter of co-production between practitioners and scientists: scientific knowledge is developed by planning, implementing, evaluating, and refining concrete interventions in concrete practices in close collaboration with these practices* (2015, p. 9).

AR has evolved over time and there are different variations of the original research design today. In contrast to the AR approach, PAR puts a stronger emphasis on the involvement of practitioners in the knowledge generation process (Cassel and Johnson 2006). Another difference is that PAR often includes well-educated experts and leaders in the research process (ibid.). PAR enables researchers to develop actionable and applied knowledge together with practitioners (Cassel and Johnson 2006).

Previous studies have shown that this methodological approach is well suited for conducting municipal climate adaptation research (e.g. Boezeman et al. 2014; Campos et al. 2016; Ruiz-Mallén 2020; Meriläinen et al. 2021; Vizinho et al. 2021). Inspired by these studies, a PAR network for the federal state of Baden-Wuerttemberg, in Germany, was initiated and established at Aalen University in 2020. The Aalen PAR network is closely tailored to regional SMCs needs, unlike the formally organised municipal networks, which have been discussed in the scientific literature for decades and which show an under-proportional participation rate of SMCs (Haupt and Coppola 2019). As part of this PAR network, a series of workshops, interviews and scientific-practical cooperation was initiated. In this research paper, the methodology and subsequent results of one workshop are discussed.

The overall goal of this joint workshop was to establish a dialogue between scientists and practitioners to find process-oriented solutions to answer the two research questions: which key actors can most effectively support adaptation measures in SMCs? And: how can a regional network for SMCs be systematically designed and established?

**Fig. 2:**
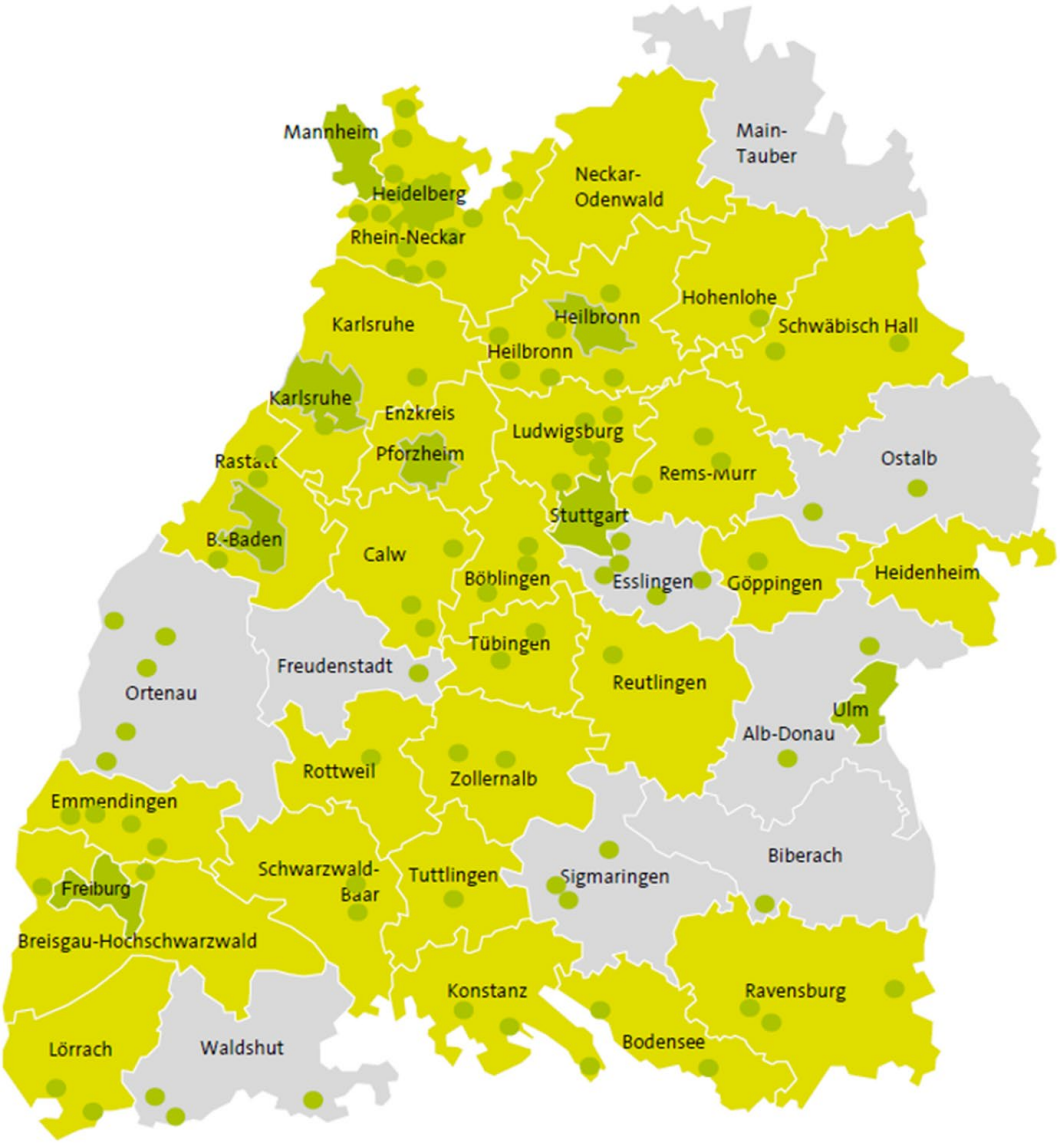
Map of the German federal state of Baden-Wuertemberg with areas of responsibility, (dark green: municipal responsibility; light green: responsibility at county level; grey: regions without registered municipal climate action managers) extract from KEA (2021)

All climate action managers registered with Baden-Wuerttemberg´s Climate Protection and Energy Agency (KEA) were invited to the workshop as participants. Municipal Climate action managers play an important role for local climate policy in German cities (Wamsler 2015; Kenkmann et al. 2021). They act as crucial interview partners for climate protection research (e.g. Zeigermann et al. 2020) and for climate adaptation research (e.g. Sprondel et al. 2016) because there are still only a few climate adaptation managers in German municipalities. Their tasks are very diverse and vary from municipality to municipality. They range from administrative work and public relations to educational projects and fundraising (Kenkmann et al. 2021). Across the state, there are about 90 climate action managers registered in the KEA network (KEA 2021). Thirty of them participated in the workshop and are altogether responsible for a total of 105 mainly small and medium-sized cities. Figure 2 illustrates their responsibilities according at the municipal level (dark green) and at the county level (light green). In the grey regions there are no climate protection managers registered with the KEA network.

Due to contact restrictions caused by the Covid-19 pandemic, the workshop took place online on December 12, 2020. The workshop was divided into a theoretical and a practical part. The theoretical part consisted of an introductory lecture and two expert lectures. In the practical part, there were two moderated electronic brainstorming sessions (EBSs, see Maaravi et al. 2020) followed by a discussion. In order to include various perspectives and generate a variety of ideas, the participants divided themselves up into three groups (beginners, practitioners and experts in climate adaptation) for each of the two EBSs. With the help of a conference tool (“Zoom”), the participants were given the opportunity to contribute their ideas and record them in writing during the EBSs. The results of the different groups were recorded by four moderators and processed both, deductively-quantitatively (for question 1) and deductively-qualitatively (for question 2). The quantitative results were presented to the participants. This was followed by a discussion and a structured summary of the results. In addition, we compared the results with those of a previous meta-study by Klein et al (2018). The qualitative evaluation was accomplished by applying the step model of inductive category development (see Mayring 2010, p. 84). This was followed by a discussion and further development of the results.

## Results

The first survey was designed to identify potential key actors for municipal climate adaptation. The participants suggested a total of 137 organisations, which have been clustered into three groups: public sector, citizens and private sector. A total of 67 ideas related to the public sector, 23 ideas related to citizens, 15 ideas related to the private sector, and 32 were duplicates or not usable. The public sector cluster included municipal offices (such as the environmental office), educational institutions (such as local universities and colleges), as well as political institutions and the health sector (with hospitals and care institutions). We sub-divided the citizens cluster into citizenship, local clubs and associations, and the private sector included regional companies, regional craftsmanship and farms.

**Fig. 3:**
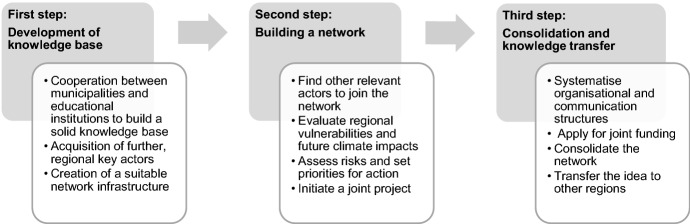
Steps for building up a regional network framework for SMCs

After defining the relevant key actors (*who?*), we examined the options for action that our participants identified (*what is to be done?*). In the second survey, the participants identified a total of 106 ideas, which we clustered into *knowledge base*-development; network-building, and consolidation and knowledge transfer. In a subsequent discussion with all participants, the (individual) results were put into a logical order. This order shows which steps are considered necessary by the participants in order to establish a regional network for SMC. Taken together, these steps form our regional network framework, which is explained step by step below (see also Fig. 3).

### First step: development of knowledge base

First of all, a consistent knowledge base, which all network members can accept and agree upon, is needed. This requires a concise, understandable and practicable presentation of scientific findings for municipalities. Regional Universities, colleges, climate research institutes and other higher education institutions were suggested as relevant key actors for developing and providing a common knowledge base. They are able to provide crucial information and support municipalities in setting up their own knowledge management systems, e.g. in creating a suitable online community platform. Complex interrelationships can thereby be presented in a simple and understandable way for stakeholder groups, particularly for citizens. The generated knowledge should be collected on the online platform and provided to the regional network actors. On this basis, measures such as information events, expert lectures and joint newsletters, as well as the conception and implementation of audio-visual climate city tours and geocaching, can be developed.

### Second step: network building

After the identification of regional key actors and the generation and implementation of an accessible knowledge base, the long-term objectives and strategy of the network have to be defined. The process can involve other relevant actors like the private sector including regional companies and local farmers. Classifying and ranking climate risks is a good starting point for climate action in the specific region where the network will be based. In the medium term, joint model projects for climate adaptation should be initiated within the network. This includes, for example, suitable heat plans with well-defined measures in the fields of greening the built urban environment, cloudburst management plans, flood plans for cities near rivers, or mobility strategies.

### Third step: consolidation and knowledge transfer

Finally, the network needs to be consolidated, which is probably the most demanding and time-consuming task. This requires organisational measures. One of these is to establish a head office for the coordination of the regional climate change adaptation network. This office has to establish and maintain effective communication structures that guarantee a continuous exchange of information and promote joint applications for funding in the longer term. Furthermore, the organisational capacity of the key actors in the network needs to be ensured by establishing staff positions for the municipal climate action managers within this office. Moreover, cooperation between the network and governmental actors at the state and federal level can also contribute to a better visibility, relevance and communication of the needs of SMCs.

### Transferability of the regional network framework

With regard to the transferability to other regions, policy makers have to consider the following issues:

- SMCs often have little experience in knowledge management and usually lack resources for generating scientific knowledge. Strategic cooperation, e.g. with local universities or colleges, can therefore play an important role (Wamsler et al. 2013; Hackenbruch et al. 2017; Bai et al. 2018). An important factor for the transferability of the framework is cooperating with potential partners that can initiate and establish an effective and adequate knowledge management.
- Even if SMCs have only limited human resources, each participating city should appoint actors responsible for climate action, to take over the role of the coordinator. External individuals can not entirely fill this role because knowledge of internal structures within the municipality is essential. The coordinators must be empowered to implement change processes in the organisation.
- Long-term cooperation between the municipal actors and the stakeholders in the network must be ensured. Short communication and flat hierarchies within the regional network are important for the ability to act proactively.
- The accessibility and quality of the information needs to be adjusted to the target group. To apply the PAR method, the level of abstraction (e.g. in communication) has to be easy to understand, rather practical and focused on action.
- In the long term, the creation of additional positions to ensure the continuity of the network is necessary. This can be achieved by an arrangement in which several municipalities share costs, and in which these new staff are responsible for the maintenance and expansion of the network.

## Discussion

By applying a PAR approach, we have developed a transferable framework for the initiation and establishment of regional networks for climate change adaptation in SMCs. Future research can address the insights and questions raised by a critical look at our results and the academic literature.

Our findings related to question 1 (*which key actors can most effectively support adaptation measures in SMCs?*) concur with previous research findings (e.g. Klein et al. 2018), as they share the categorisation of different stakeholders into public sector, private sector and citizens. In their meta study of adaptation initiatives, Klein et al. saw the greatest potential in the public sector, followed by citizens` initiatives. However, the climate action managers interviewed in our study saw greater potential in citizens than in the private sector. An explanation might be that the municipal climate action managers who participated in our workshop generally have more and stronger interactions with citizens´ groups in their daily work than they do with businesses.

With regard to stakeholders for municipal climate adaptation in Baden-Wuerttemberg, Hackenbruch et al. (2017) recommend stronger cooperation between municipalities and climate researchers. At the same time, Hackenbruch et al. note that free scientific climate information services (e.g. online on the website of the Federal Environmental Agency (UBA), or federal state level (LUBW)) are used rarely by municipalities. This suggests that direct cooperation with municipalities, like e.g. within the Aalen PAR network, are a feasible way to exchange information. However, we cannot assume that this applies to all municipalities, because networking is time-consuming and only a fraction of the municipalities in Baden-Württemberg participated in the workshop. Furthermore, PAR approaches outside of Baden-Wuerttemberg might also lead to (more or less) different results. Whether the results are fully applicable to other regions or even to other German federal states might be questioned. Indeed, German states are relatively autonomous in supporting (or not supporting) their municipalities to prepare for climate change. In order to formulate robust statements about the transferability potential of our framework we need more regional case studies applying a PAR approach.

The previous points dealt mainly with network partners and network building. In addition to these plans, however, it is also important to be aware of the considerations that become important after a loss event. Several scholars emphasise the potential of social ties among residents across locations for preparedness and reconstruction after environmental threats (Rockenbauch and Sakdapolrak 2017; Patterson et al. 2019; Wilkin et al. 2019). Cope et al. (2018) show that in the US American state of Louisiana, which was hit by disasters quite frequently, residents with strong ties to individuals two or more hours away from their community perceive themselves to be better prepared and adequately resourced to cope with environmental threats than individuals with strong ties in their immediate local community.

Our work considered the cooperation of actors within the federal state of Baden-Wuerttemberg. As described in the case selection, Baden-Wuerttemberg has different geographical landscapes with different climate risks, such as increasingly hot and dry summers (Muthers et al. 2017), hail- and thunderstorms (Puskeiler et al. 2016) or floods (Hennegriff et al. 2006). However, due to its size and variety of landscape, it is unlikely that a single extreme weather event such as a flood or storm will affect the entire state. Moreover, not all municipalities are equally affected by all climate change risks. Thus, for their further development and growth, networks for SMCs should not necessarily stop at regional or national borders and rather be conceptualised and planned on a supra-regional basis, taking into account climate risks and existing institutional and administrative settings. It is therefore conceivable to extend our network to neighbouring states such as Bavaria and the neighbouring countries such as Switzerland and Austria. There is high potential in this expansion, because, geographically speaking, the climate impact will be similar. Moreover, a regional network can be sub-divided into different working-groups focussing on certain climate risks.

## Conclusion

Climate networks of predominantly large cities have existed since the 1990s (Labaeye and Sauer 2013). In contrast, regional networks for SMCs are far less numerous and visible. Nevertheless, in many regions worldwide large parts of the population live in SMCs (Giffinger et al. 2007; Bell and Jayne 2009). Through its methodical application of a PAR approach this research seeks to provide SMCs with a regional network framework, i.e. an easily transferable and manageable tool for building regional networks. To achieve this, we found practical ideas and solutions in close cooperation with the climate protection managers of the state. Our study demonstrates how to identify key actors who can support municipal adaptation practice. For the case of Baden-Wuerttemberg, the most important actors were in public sector organisations, followed by citizens and the private sector. Strategic cooperation with educational institutions such as universities and colleges plays an important role within the public sector. Based on these findings, we demonstrated how a network for SMCs can be built up systematically. The framework developed in the course of this work consists of three steps: developing the knowledge base; building the network; and consolidating and transferring knowledge to further cases. It fills the gap of regionally and locally tailored solutions for SMCs (Granberg et al. 2019) and contributes to climate adaptation research for SMCs in general. Nevertheless, much more research on how SMCs can successfully tackle local climate adaption challenges is urgently needed. Indeed, SMCs are already affected by the consequences of climate change, and will be affected even more in the future (Drolet and Sampson 2017; Dannenberg et al. 2019). Moreover, they lack the capacity (financial resources and staff) to pioneer climate adaptation policies and do not receive the same (scientific) attention as larger and more-internationally oriented cities (Kern 2019). Given these disadvantages, SMCs need to pool their resources. By joining forces through regional networks SMCs can have the opportunity to exchange crucial information on municipal climate adaptation practice and learn with and from each other.

## Data Availability

Not applicable.
